# PL201, a Reported Rhamnoside Against Alzheimer's Disease Pathology, Alleviates Neuroinflammation and Stimulates Nrf2 Signaling

**DOI:** 10.3389/fimmu.2020.00162

**Published:** 2020-02-27

**Authors:** Yuqian An, Hong Zhang, Shichao Huang, Gang Pei

**Affiliations:** ^1^State Key Laboratory of Cell Biology, CAS Center for Excellence in Molecular Cell Science, Shanghai Institute of Biochemistry and Cell Biology, Chinese Academy of Sciences, University of Chinese Academy of Sciences, Shanghai, China; ^2^Shanghai Key Laboratory of Signaling and Disease Research, Collaborative Innovation Center for Brain Science, School of Life Sciences and Technology, Tongji University, Shanghai, China; ^3^Institute for Stem Cell and Regeneration, Chinese Academy of Sciences, Beijing, China

**Keywords:** Alzheimer's disease, neuroinflammation, microglia, rhamnoside analog, nuclear factor erythroid 2-related factor 2, heme oxygenase 1, nuclear factor kappa B

## Abstract

Neuroinflammation induced by overactivated glia cells is believed to be a major hallmark of Alzheimer's disease (AD) and a hopeful target against AD. A rhamnoside PL201 was previously reported to promote neurogenesis and ameliorate AD, and in this study, we revealed that PL201 also significantly reduced accumulation of the activated microglia and proinflammatory cytokines in APP/PS1 mice. *In vitro*, PL201 consistently suppressed the microglia induction of proinflammatory cytokines after stimulation with lipopolysaccharides and Aβ42. Further mechanistic studies demonstrated that PL201 considerably enhanced the expression level and the nuclear translocation of Nrf2, a key regulator of neuroinflammation. Moreover, PL201 effectively stimulated Nrf2 signaling cascade, including upregulation of HO-1 and downregulation of NF-κB pathway. Thus, our findings indicated the anti-neuroinflammatory effect by PL201 *in vivo* and suggested that PL201 or the like, with multiple functions such as neurogenesis, mitochondria maintenance, and anti-neuroinflammation, could be a promising candidate in AD treatment.

## Introduction

Alzheimer's disease (AD) is a chronic neurodegenerative disease and the most common cause of dementia (1). Mounting findings suggest a significant role of neuroinflammation in the pathogenesis of AD (2–4). Microglia, which are the major resident immune cells in the central nervous system (CNS), play a critical role in regulating neuroinflammation (5, 6). As a prominent feature of AD, microglia are recruited to amyloid beta (Aβ) plaques and being activated (7–9). Then, these activated microglia release chemokines, cytokines, and other inflammatory mediators such as IL-1β, IL-6, TNFα, MIP-1α, inducible nitric oxide synthase (iNOS), and cyclooxygenase-2 (COX-2), which will further aggravate neuronal damage and AD pathologies (2, 10–12).

Accumulating evidence suggest that nuclear factor erythroid 2-related factor 2 (Nrf2) is a transcription factor that critically involved in regulating the inflammatory response of microglia (13–15). Mechanistic studies reveal that, upon proper stimulation, Nrf2 translocates into the nucleus and activates its downstream targets including Heme oxygenase 1 (HO-1) (15–18). The upregulation of Nrf2 further inhibited NF-κB signaling to exert anti-inflammatory effects (19, 20). The initiation of this process is tightly regulated by Kelch-like ECH-associated protein 1 (Keap1)-mediated Nrf2 ubiquitination and degradation (21). Increasing lines of evidence reveal that Nrf2/HO-1 pathway is dysregulated in AD (22–27). For example, Ramsey et al. reported that the expression of nuclear Nrf2 was decreased in the hippocampi of AD patients (23). Similarly, Kanninen et al. found that overexpression of Nrf2 in the hippocampus reduces abnormal glia activation in a mouse model of AD (25). These findings indicated that proper Nrf2 signaling activators may serve as potential therapeutic interventions for AD.

AD is a multifactorial disease. Since their structural complexity and multitarget activity, natural products and their derivatives are recognized as an important source for developing AD drug leads (28–30). For instance, curcumin and its derivatives have been reported to ameliorate multiple AD pathologies including inhibiting Aβ production and tau phosphorylation, reducing neuroinflammation and oxidative stress, promoting neural stem cell proliferation and adult neurogenesis, and reversing cognitive deficits (31–33). Rhamnoside is a common kind of naturally occurring glycoside with antiaging effects. Moreover, it has also been revealed that rhamnoside may exert anti-AD effects by inhibiting production and aggregation of Aβ42 (34). We previously reported that a designed compound PL201, which was derived from rhamnoside, promoted neurogenesis and ameliorated cognitive impairment in the APP/PS1 mice (35). In this study, we found that PL201 significantly reduced microglia accumulation and proinflammatory cytokine production in the APP/PS1 mice. We also demonstrated that PL201 effectively suppressed LPS and Aβ induced proinflammatory cytokine expression in BV2 and human microglia cells. Furthermore, we elucidated that PL201 may exert its anti-inflammatory effect through Nrf2/HO-1/NF-κB signaling. In conclusion, PL201 may be served as a promising multitarget drug candidate for the treatment of AD.

## Methods

### Animals

All animal studies were carried out in accordance with the guidelines of the Institutional Animal Care and Use Committee (IACUC) of the Institute of Biochemistry and Cell Biology, Shanghai Institutes for Biological Sciences, Chinese Academy of Sciences. Both male and female mice were randomized in different experiment groups in this study. Nine-month-old APP/PS1 mice and wild-type (WT) littermates were used in this study. APP/PS1 transgenic mice expressing the mutated human app (Swedish mutations K595N M596L) and the human presenilin1 with a deletion of exon 9 were obtained from The Jackson Laboratory (stock no.004462) (36). All mice were bred and maintained in a temperature-controlled room and maintained on a 12-h light/dark cycle, with *ad libitum* access to food and water.

### Drug Administration

PL201 was synthesized as previously described (35). PL201 was dissolved in water. Nine-month-old APP/PS1 mice were orally administered with 30 mg/kg PL201 daily for 60 days. Control mice were treated with water as a vehicle. After drug treatment, mice of each group were euthanized, and the whole brains were removed. Brains were collected for measurement of proinflammatory and anti-inflammatory cytokines.

### Cell Culture and Drug Treatment

BV2 cells were cultured in DMEM (Invitrogen, Camarillo, CA); HMC3 cells were cultured in MEM (Invitrogen, Camarillo, CA). All medium was supplemented with 10% heat-inactivated fetal bovine serum (FBS), 100 U/ml penicillin, and 100 μg/ml streptomycin, and all cells were cultured at 37 °C in an atmosphere of 95% air and 5% CO_2_.

In all *in vitro* assays, BV2 and HMC3 cells were starved for 12 h in serum-free culture medium before PL201 treatment. To detect the expression of proinflammatory cytokines, cells were pretreated with PL201 at various concentrations (10–300 μM). After the pretreatment, 300 ng/ml LPS (Sigma, O55:B5) or 3 μM oligomer Aβ42 (GL Biochem, Shanghai) was added for further 24 h stimulation. To detect Nrf2′s downstream genes, cells were treated with PL201 at various concentrations (10–300 μM) for 6 h. To identify the nuclear translocation and expression of Nrf2, cells were treated with PL201 at various concentrations (10–300 μM) for 1 h. To assess the activation of NF-κB pathway, cells were pretreated with PL201 for 2 h followed by 1 h of LPS or oligomer Aβ42 stimulation.

### Preparation of Aβ42 Oligomers

Aβ42 peptide was prepared as previously described (37). In brief, 1 mg Aβ42 was dissolved in 1 ml cold hexafluoroisopropanol (Sigma, 52517), and the solution was aliquoted into Protein LoBind tubes (Eppendorf, 030108094); each tube containing 50 μg Aβ42 was dried overnight at RT. The residue was dissolved in 2.5 μl dimethyl sulfoxide (DMSO) followed by 117 μl cold phenol-free DMEM/F-12 medium (Thermo Fisher, 21041025) to obtain a 100-μM stock solution. After vortex for 60 s and incubated at 4 °C for 24 h, the solution was centrifuged at 16,000 × g for 10 min, and the supernatant containing Aβ42 oligomers was used for further experiments. The content of Aβ42 oligomers was confirmed by Western blot using anti-Aβ antibody (6E10, BioLegend, 803002; 1:1,000).

### Preparation of Mouse Brain Homogenate

Mice cortex extract was obtained as reported previously (38, 39). In brief, cortex tissues were homogenized and sonicated 30 × 15 s with a 15-s interval between each sonication in Tris-buffered saline (TBS, pH 8.0) containing 0.1% Igepal. Samples were centrifuged at 16,000 × g at 4 °C for 30 min to remove any remaining insoluble debris in the solution. Samples were then detected by ELISA kits. The results were normalized to total protein.

### Enzyme-Linked Immunosorbent Assay

The ELISA experiments were performed following manufacturer's instructions including the following: mouse TNFα (DAKEWE, 1217202), mouse IL-6 (DAKEWE, 1210602), mouse IL-1β (DAKEWE, 1210122), mouse MCP-1 (DAKEWE, 1217392), mouse IL-10 (DAKEWE, 1211002), and mouse MIP-1α (NEOBIOSCIENCE, EMC010a).

### Quantitative Real-Time Polymerase Chain Reaction

Total RNA was extracted from BV2 cells and HMC3 cells using TRIZOL Reagent according to the manufacturer's protocols. Equal amounts of RNA were reverse transcribed using a cDNA synthesis kit (Takara). qRT-PCR experiments were performed using synthetic primers and a 2 × HotStart SYBR Green qRT-PCR Master Mix kit (ExCell). Primers used in this study are listed in Table 1.

**Table 1:** Primers used for qRT-PCR.

**Primer**	**Forward(5′-3′)**	**Reverse(5′-3′)**
Mouse IL-1β	AAGCCTCGTGCTGTCGGACC	TGAGGCCCAAGGCCACAGGT
Mouse IL-6	GCTGGTGACAACCACGGCCT	AGCCTCCGACTTGTGAAGTGGT
Mouse TNFα	CAAGGGACAAGGCTGCCCCG	GCAGGGGCTCTTGACGGCAG
Mouse MCP-1	TTAAAAACCTGGATCGGAACCAA	GCATTAGCTTCAGATTTACGGGT
Mouse MIP-1α	TGTACCATGACACTCTGCAAC	CAACGATGAATTGGCGTGGAA
Mouse iNOS	CAGCTGGGCTGTACAAACCTT	CATTGGAAGTGAAGCGTTTCG
Mouse COX-2	GCAAATCCTTGCTGTTCCAACC	GGAGAAGGCTTCCCAGCTTTTG
Mouse IL-4	GGTCTCAACCCCCAGCTAGT	GCCGATGATCTCTCTCAAGTGAT
Mouse IL-10	GCTCTTACTGACTGGCATGAG	CGCAGCTCTAGGAGCATGTG
Mouse CD206	CTCTGTTCAGCTATTGGACGC	TGGCACTCCCAAACATAATTTGA
Mouse HO-1	AGCAGGACATGGCCTCT	TCTGTCAGCATCACCTGCAG
Mouse NQO1	AGGATGGGAGGTACTCGAATC	TGCTAGAGATGACTCGGAAGG
Mouse Nrf2	CAGTGCTCCTATGCGTGAA	GCGGCTTGAATGTTTGTC
Mouse HO-2	AGCACATGACCGAGCAGAAAA	GCTCCGTGGGGAAATATAAGGG
Mouse GCLM	AGGAGCTTCGGGACTGTATCC	GGGACATGGTGCATTCCAAAA
Human IL-1β	AGCTACGAATCTCCGACCAC	CGTTATCCCATGTGTCGAAGAA
Human TNFα	TCTTCTCGAACCCCGAGTGA	CCTCTGATGGCACCACCAG
Human MCP-1	GTGCAGAGGCTCGCGAGCTA	CAGGTGGTCCATGGAATCCTG
Human iNOS	TCCGAGGCAAACAGCACATTCA	GGGTTGGGGGTGTGGTGATGT
Human COX-2	TCCACAGTTACCCGGAGTTTA	GCCGAGCTATCAACCGGAT
Human IL-6	TGCACTTTATGACGCACTCAC	TGTCCAAAAACACGAAATCATGC
Human HO-1	AAGACTGCGTTCCTGCTCAAC	AAAGCCCTACAGCAACTGTCG
Human HO-2	CCACCACGGCACTTTACTTCA	CGTTCTGCCCTATGTAGTGGA
Human Nrf2	TCCGGGTGTGTTTGTTCCAA	CGCCCGCGAGATAAAGAGTT

### Immunostaining and Confocal Microscopy

Immunostaining was performed as previously described (28). In detail, mice were killed, and dissected brains were fixed in 4% paraformaldehyde (PFA) (Sigma) at 4 °C for 24 h. After fixation, tissues were washed in cold PBS for three times and then dehydrated in 30% sucrose overnight at 4 °C and embedded in optimal cutting temperature (OCT) (Thermo Fisher), followed by continuous serial cryostat sectioning at 30 μm. Sections were then washed with PBS (pH 7.4) and blocked with a blocking buffer (PBS supplemented with 0.5% Triton X-100 and 5% normal horse serum) for 1 h at room temperature. Afterwards, the sections were incubated with the following primary antibodies overnight at 4 °C: anti-Iba1 (Wako, 019-19741; 1:500), and anti-6E10 (BioLegend, 803002, 1:300). These sections were washed with PBS and incubated with Alexa fluorescence-conjugated secondary antibodies (Invitrogen, 1:1,000) for 1 h at room temperature followed by 10-min nuclei staining using 4′6-diamidino-2-phenylindole (DAPI Beyotime Biotechnology, 1:5,000). The images were acquired using a Zeiss confocal laser scanning microscope (Axio Scan.Z1). The instrument settings, including numeric gain, laser power, and magnification, were kept constant between animals to avoid potential technical artifacts.

Cultured BV2 cells and HMC3 cells were fixed in 4% PFA in PBS at room temperature for 15 min, and the immunostaining was carried out following the same procedure as brain sections. The following primary antibodies were used: anti-p65 (Santa Cruz, sc-372; 1:300) and anti-Nrf2 (Cell Signaling, 12721; 1:200). The images were captured using a Zeiss confocal laser scanning microscope (Zeiss 880 Airyscan). The instrument settings, including numeric gain, laser power, and magnification, were kept constant between animals to avoid potential technical artifacts.

As previously reported, ImageJ software was applied for quantitative analysis for Iba1 staining (39). First, red/green/blue (RGB) images were converted to 8-bit grayscale, and the threshold levels were adjusted to highlight positive signals. We quantified at least four sections per mouse. The Iba1 intensity from the cortex and hippocampus were qualified via Image J. Statistical analysis based on a blind review of data.

ImageJ software was applied for quantitative analysis for Nrf2 and p65 in cultured BV2 cells and HMC3 cells. The quantitative method was used following the same procedure as brain sections.

### Western Blot Analysis

Western blotting were performed using the standard sodium dodecyl sulfate polyacrylamide gel electrophoresis (SDS-PAGE) method as previously reported (40). In brief, cultured BV2 and HMC3 cells were lysed using the protein lysis buffer (Sigma, R0278), and each sample containing 20 μg of total protein was electrophoresed with 8 or 10% SDS-PAGE. Then, these samples were transferred to nitrocellulose filter membranes (GE Healthcare Life science, A13711263). These membranes were blocked with 5% (*w*/*v*) skimmed milk for 1 h at room temperature. After that, the membrane was incubated with the following primary antibodies overnight at 4 °C: anti-iNOS (Cell Signaling, 13120S; 1:1,000), anti-COX-2 (Cell Signaling, 12282T; 1:1,000), anti-p65 (Santa Cruz, sc-372; 1:1,000), anti-p50/p105 (Beyotime Institute of Biotechnology, AF1246; 1:1,000), anti-Phospho-IκBα (Ser32) (Cell Signaling, 2859T; 1:1,000), anti-IκBα (Cell Signaling, 4814T; 1:1,000), anti-HO-1 (ABclonal, A1346; 1:1,000), anti-HO-2 (ABclonal, A2745; 1:1,000), anti-GCLM (ABclonal, A5314; 1:1,000), anti-NQO1 (Abclonal, A19586, 1:1,000), anti-Nrf2 (Cell Signaling, 12721; 1:1,000), anti-PCNA (Cell Signaling, 13110S, 1:1,000), and anti-Actin (Cell Signaling, 3700S, 1:1,000). After being washed with Tris-buffered saline with Tween 20 (TBST), these membranes were incubated with the secondary antibodies for 1 h at room temperature. The proteins of interest were visualized using an ECL Western blot detection kit (Bio-Rad). ImageJ software was applied to evaluate the densitometry. Actin or proliferating cell nuclear antigen (PCNA) were used as loading controls.

### Nuclear and Cytoplasmic Extraction

BV2 cells and HMC3 cells were cultured in six-well plates and grown for 24 h and then treated with various concentrations of PL201 for indicated time. Then, these cells were washed with PBS and lysed for 10 min in buffer A (10 mM Tris–HCl, pH 7.5, 10 mM NaCl, 3 mM MgCl_2_, and 1% NP-40) containing phosphatase inhibitor and protease inhibitor. After centrifugation at 3,000 × g for 5 min at 4 °C, the supernatant was collected to a new microfuge tube as the cytoplasmic fraction. The pellet was washed in buffer A for three times and resuspended in buffer B (20 mM HEPES, pH 7.9, 20% glycerol, 1.5 mM MgCl_2_, 0.2 mM EDTA, 1 mM dithiothreitol, and 1 mM phenylmethylsulfonyl fluoride). The suspension was then sonicated for 30 s, and centrifuged at 14,000 × g for 10 min at 4 °C to obtain the nuclear fraction.

### Cell Viability

Cell viability was detected by CellTiter-Glo Luminescent assay. One hundred microliters CellTiter-Glo Reagent was added to each well of 96-well plate and mixed for 2 min on an orbital shaker to lyse cells. The cell viability was judged according to the luminescence.

### Statistical Analysis

GraphPad Prism 7 (GraphPad Software, La Jolla, CA, United States) was used to draw graphs and perform data analysis. All values were represented as the mean ± SEM. One-way ANOVA followed by the Tukey multiple comparisons test were used to compare values from multiple groups. Two-tailed unpaired *t*-test was applied for comparisons between two groups. Values were considered to have significant difference when *p* < 0.05.

## Results

### PL201 Treatment Decreases Microglia Reactivity and Proinflammatory Cytokine Production in APP/PS1 Mice

**Figure 1:**
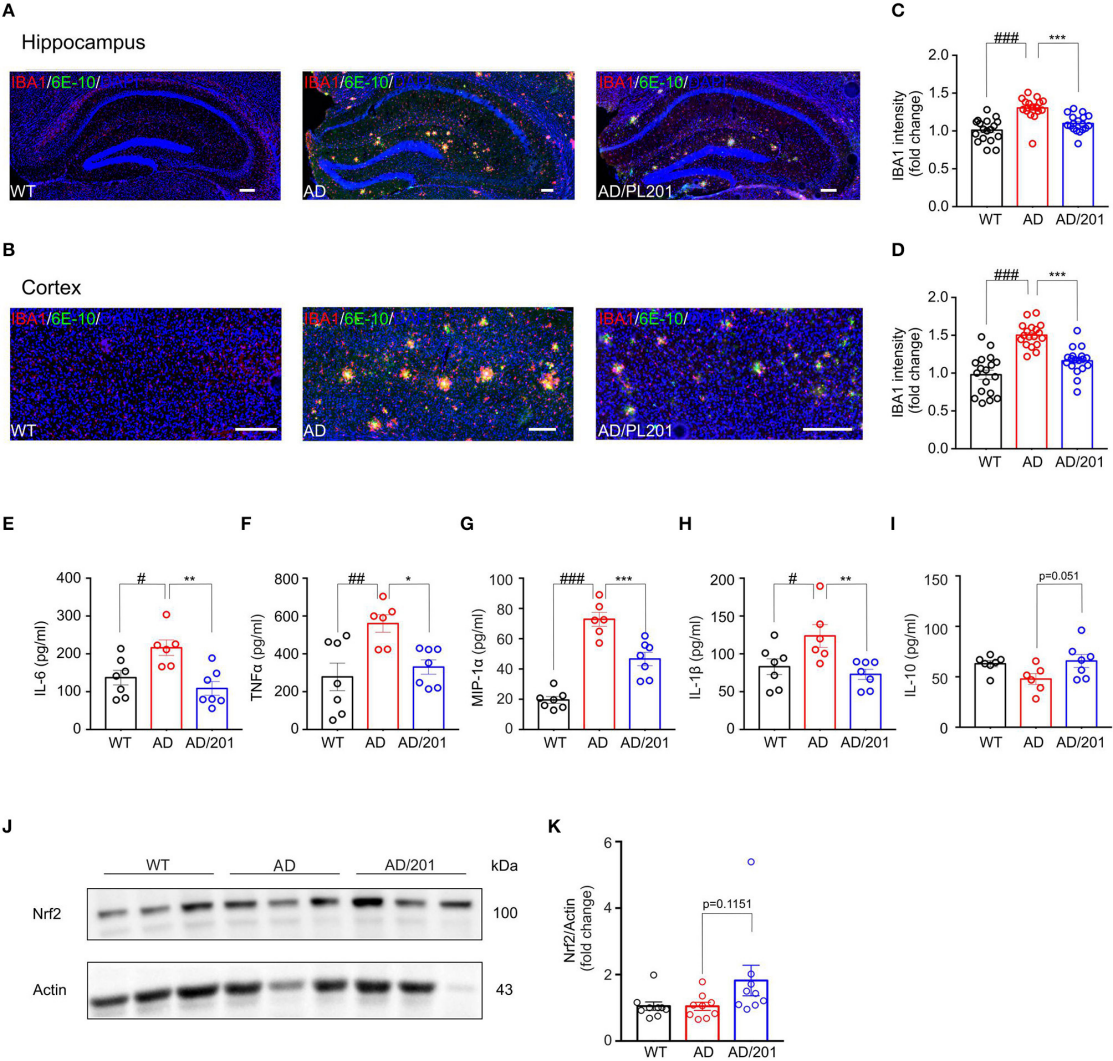
PL201 treatment decreases microglia reactivity and proinflammatory cytokine production in APP/PS1 mice. **(A–D)** Brain sections of WT, AD, and PL201 treated AD mice were immunostained with the microglial markers Iba1. **(A,B)** Representative images of Iba1 staining of hippocampus regions and cortex regions from WT, AD, and PL201 treated AD mice. Scale bar = 100 μm. **(C,D)** Quantification of Iba1 intensity from sections. *n* = 4 mice per group. **(E–I)** Indicated cytokine or chemokine levels in mouse brain were detected by ELISA. *n* = 7 (WT), 6 (AD), and 7 (AD/PL201) mice. **(J,K)** Nrf2 activation in mouse brains were detected by Western blot. Quantifications were expressed as mean ± SEM (compared with WT, ^#^*p* < 0.05, ^##^*p* < 0.01, ^###^*p* < 0.005; compared with AD mice, *p < 0.05, **p < 0.01, ***p < 0.005).

Accumulating evidence suggest that the pathogenesis of Alzheimer's disease is strongly associated with immunological alterations in the brain. In AD patients and mouse models, neuroinflammation has often been observed accompanied by excessive accumulation of microglia. To investigate whether PL201 treatment affected neuroinflammation in AD mice, we evaluated the microglial reactivity in the hippocampus and cortex by staining with ionized calcium binding adaptor molecule 1 (Iba1) antibody, which is a specific marker of microglia in the brain. We found that the microglia accumulating around the amyloid plagues significantly increased in the hippocampus and cortex of APP/PS1 mice when compared with the WT controls (Figures 1A,B). Moreover, reactive microgliosis was reduced in PL201-treated AD mice as well (Figures 1A–D). Since the activated microglia produce cytokines and chemokines, which contribute to AD pathology, we further detected the level of several cytokines and chemokines in brain samples from WT and AD mice. The results showed that, in AD mice, the levels of proinflammatory cytokines and chemokines including IL-6, TNFα, MIP-1α, and IL-1β were elevated, while the level of anti-inflammatory cytokine IL-10 was decreased (Figures 1E–I). After PL201 treatments, the levels of these proinflammatory cytokines were reduced in AD mice, but the levels of IL-10 remained unchanged. Collectively, these data demonstrated that PL201 treatment could suppress proinflammatory cytokine secretion and decrease neuroninflammation in AD mice. Emerging findings suggest that a significant increase in inflammatory response in AD is associated with Nrf2. We examined Nrf2 expression in mouse brains and found that PL201 had a tendency to increase Nrf2 expression in AD mice (Figures 1J,K).

### PL201 Attenuates LPS-Induced Proinflammatory Factor Release *in vitro*

**Figure 2:**
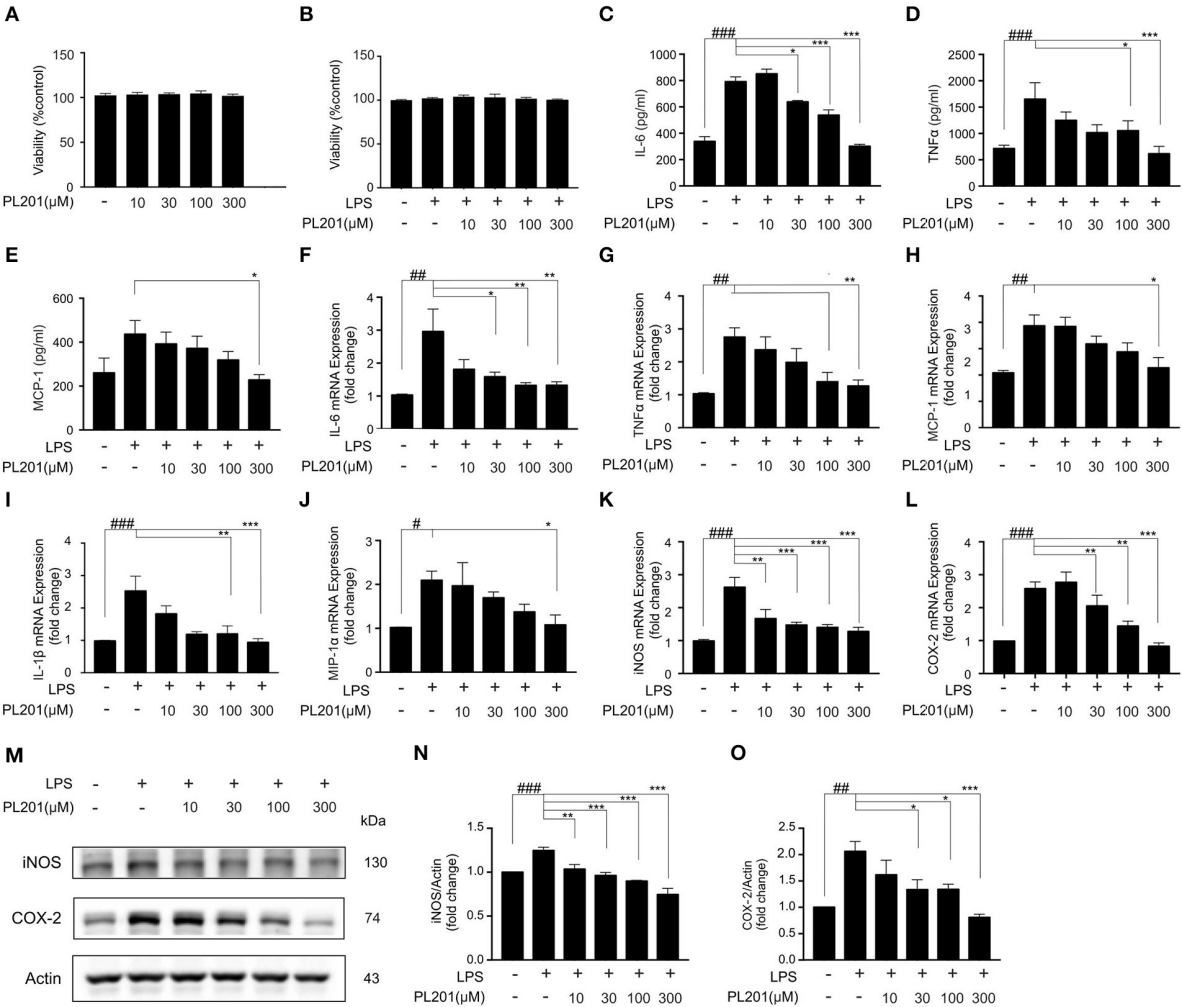
PL201 attenuates LPS-induced proinflammatory factor release *in vitro*. **(A,B)** PL201 has no cytotoxicity on BV2 cells. **(C–O)** BV2 cells were pretreated with PL201 for 2 h followed by LPS stimulation for further 24 h. **(C–E)** The levels of IL-6, TNFα, and MCP-1 in culture supernatant were measured by ELISA. **(F–L)** The mRNA expressions of IL-6, TNFα, MCP-1, IL-1β, MIP-1α, iNOS, and COX-2 were measured by qRT-PCR. **(M–O)** Representative Western blot analysis of iNOS and COX-2. Quantifications were expressed as mean ± SEM (compared with control, ^#^*p* < 0.05, ^##^*p* < 0.01, ^###^*p* < 0.005; compared with LPS-stimulated condition, *p < 0.05, **p < 0.01, ***p < 0.005).

Having established that PL201 decreases neuroinflammation in AD mice, we next tested the direct effects of PL201 on microglia cells *in vitro*. First, we examined the cytotoxicity of PL201. Twenty-Four hours treatment of PL201 (10–300 μM) exhibited no toxicity in BV2 cells (Figures 2A,B). Then, we investigated the effect of PL201 on LPS-induced cytokines and chemokines release. After pretreatment for 2 h with PL201, BV2 cells were exposed to LPS for 24 h (Figures 2C–O). We found that PL201 dose dependently inhibited the production of proinflammatory cytokines including IL-6, TNFα, and MCP-1, which were induced by 300 ng/ml LPS (Figures 2C–E). Similarly, PL201 effectively reduced the LPS-induced mRNA expression of IL-6, TNFα, MCP-1, IL-1β, MIP-1α, iNOS, and COX-2 (Figures 2F–L). PL201 also decreased protein level of iNOS and COX-2 in a concentration-dependent manner (Figures 2M–O). Besides, we tested the effect of PL201 on the expression profile of anti-inflammatory cytokines including IL-4, IL-10, and CD206; no statistically significant change was observed (Figures S1A–C). In AD, Aβ stimulates the microglia to aggravate neuroinflammation. Therefore, in the present study, we stimulated HMC3 human microglia cells with the soluble oligomer Aβ42, and then tested whether PL201 could exert similar anti-inflammatory effect. The results showed that PL201 also significantly reduced the levels of Aβ42-induced proinflammatory cytokines including IL-6, TNFα, MCP-1, IL-1β, iNOS, and COX-2 (Figures S2A–H).

### PL201 Activates Nrf2 Signaling

**Figure 3:**
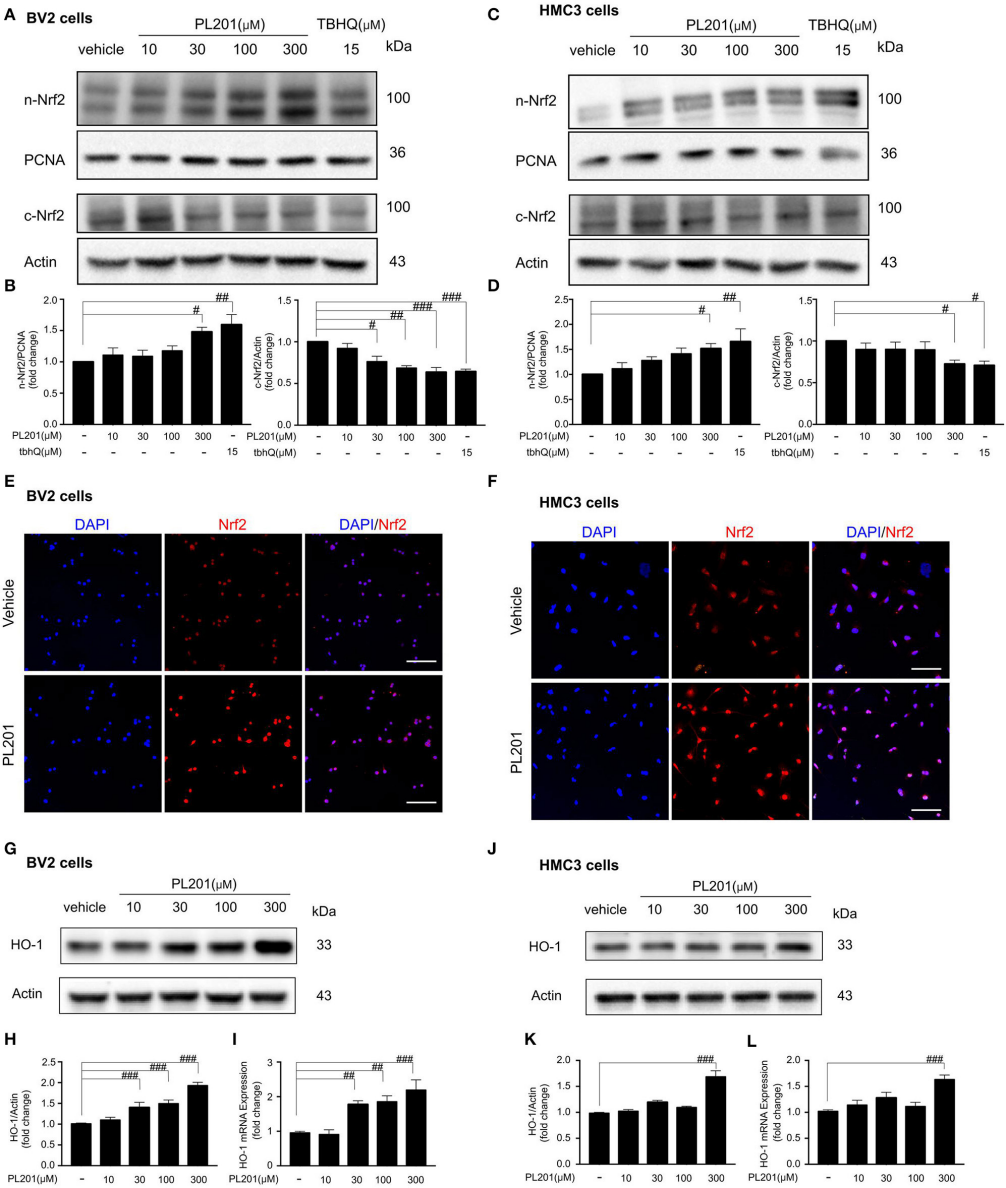
PL201 activates Nrf2 signaling. **(A,B)** The expression of Nrf2 in nuclear and cytoplasmic fractions from BV2 cells treated with various concentrations of PL201 for 1 h was measured via Western blot. tbhQ as a positive control. **(C,D)** The expression of Nrf2 in nuclear and cytoplasmic fractions from HMC3 cells treated with various concentrations of PL201 for 1 h was measured via Western blot. tbhQ as a positive control. **(E)** BV2 cells were pretreated with PL201 for 1 h; the nuclear translocation of Nrf2 was detected by immunostaing. Scale bar = 100 μm. **(F)** HMC3 cells were pretreated with PL201 for 1 h; the nuclear translocation of Nrf2 was detected by immunostaing. Scale bar = 100 μm. **(G–I)** BV2 cells were treated with PL201 for 6 h, representative Western blot analysis of HO-1 and mRNA expression of HO-1. **(J–L)** Representative Western blot analysis of HO-1 and mRNA expression of HO-1 in HMC3 cells were treated with PL201 for 6 h. Quantifications were expressed as mean ± SEM (compared with control, ^#^*p* < 0.05, ^##^*p* < 0.01, ^###^*p* < 0.005).

Since Nrf2 signaling plays an important role in modulating neuroinflammation, we next tested whether PL201 could activate Nrf2 signaling in both BV2 and HMC3 cells. The results revealed that PL201 activated Nrf2 nuclear translocation in a dose-dependent manner. Meanwhile, Nrf2 levels in the cytoplasm were correspondingly reduced after PL201 treatment (Figures 3A–D). To detect the specificity of Nrf2 antibody (41), we used tbhQ (15 μM) as a positive control and showed the whole extension of lanes in the Western blot. (Figures S3A,B). Similarly, using immunostaining, we observed that nuclear accumulation of Nrf2 was markedly increased after 1 h treatment with PL201 in BV2 (Figure 3E) and HMC3 cells (Figure 3F). We also determined gene level of Nrf2 after PL201 stimulation; the results indicated that PL201 effectively activated the mRNA expression of Nrf2, with a significant effect at the concentration of 300 μM in BV2 and HMC3 cells (Figures S3C,D). HO-1 is a major Nrf2 target gene; therefore, we tested the level of HO-1. The expression of the HO-1 significantly increased after PL201 treatment for 6 h in BV2 cells (Figures 3G–I). Similar result was also observed in HMC3 cells (Figures 3J–L). Moreover, the function of Nrf2 in the regulation of HO-1 induced by PL201 was studied using Nrf2 special inhibitor—brusatol. BV2 cells were pretreated with brusatol followed by PL201 for 6 h; brusatol completely abolished PL201-induced HO-1 expression (Figures S3E,F). Interestingly, we tested HO-2 in parallel to HO-1 and found that the protein level of HO-2 was not altered after PL201 treatment (Figures S4A–F). Besides HO-1, 6 h treatment of PL201 also dose-dependently increased the levels of GCLM and NQO1, which are both NRF2-target genes (Figures S5A–F). Taken together, these results indicated that PL201 treatment activates Nrf2 signaling pathway.

### Nrf2 Signaling Is Required for the Anti-inflammatory Effects of PL201

**Figure 4:**
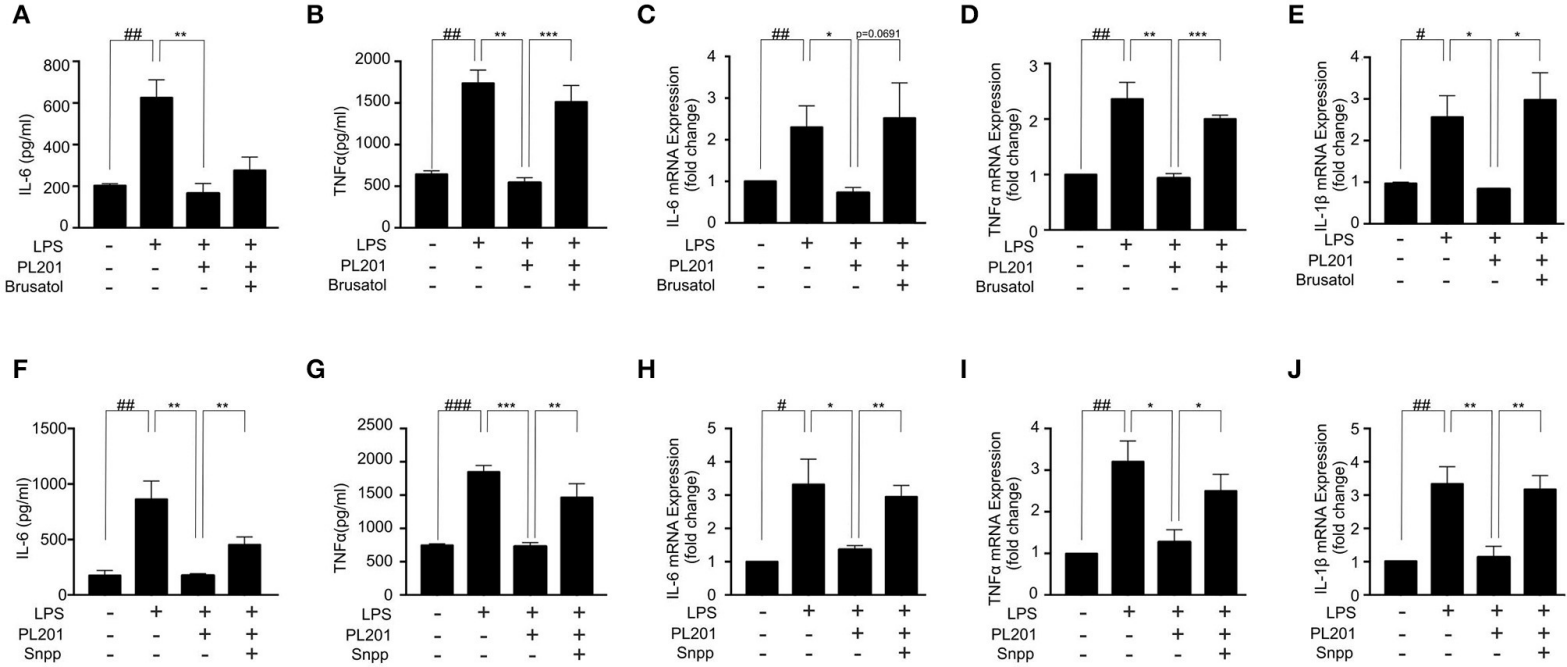
Nrf2 signaling is required for the anti-inflammatory effects of PL201. **(A–E)** BV2 cells were treated with PL201 in the presence or absence of brusatol (30 nM) and then exposed to LPS for 24 h. **(A,B)** The levels of IL-6 and TNFα in culture supernatant were measured by ELISA. **(C–E)** The mRNA expression of IL-6, TNFα, and IL-1β were measured by qRT-PCR. **(F–J)** BV2 cells were treated with PL201 in the presence or absence of Snpp (10 μM) and then exposed to LPS for 24 h. **(F,G)** The levels of IL-6 and TNFα in culture supernatant were measured by ELISA. **(H–J)** The mRNA expression of IL-6, TNFα, and IL-1β were measured by qRT-PCR. Quantifications were expressed as mean ± SEM (compared with control, ^#^*p* < 0.05, ^##^*p* < 0.01, ^###^*p* < 0.005; compared with LPS-stimulated condition, *p < 0.05, **p < 0.01, ***p < 0.005).

We next investigated whether the anti-inflammatory effects of PL201 were mediated by Nrf2 signaling. To test this, we pretreated BV2 cells with an Nrf2-specific inhibitor brusatol before adding PL201 and found that brusatol attenuated the inhibitory effect of PL201 on the release of proinflammatory cytokines in LPS-treated BV2 cells (Figures 4A–E). We next blocked HO-1, which is a critical downstream mediator of Nrf2, by its inhibitor SNPP. BV2 cells were pretreated in the absence or presence of SNPP or PL201, followed by LPS stimulation for 24 h. Similar to that of Nrf2 inhibition, SNPP also attenuated the inhibitory effects of PL201 on proinflammatory cytokines such as IL-6, TNFα, and IL-1β (Figures 4F–J). We investigated the effect of PL201, brusatol, and Snpp on cytokines expression alone. They did not have effect on the expression of proinflammatory cytokines (Figures S6A–C). In summary, these data suggested that Nrf2 signaling is required for the anti-inflammatory effects of PL201.

### PL201 Suppresses NF-κB Pathway

**Figure 5:**
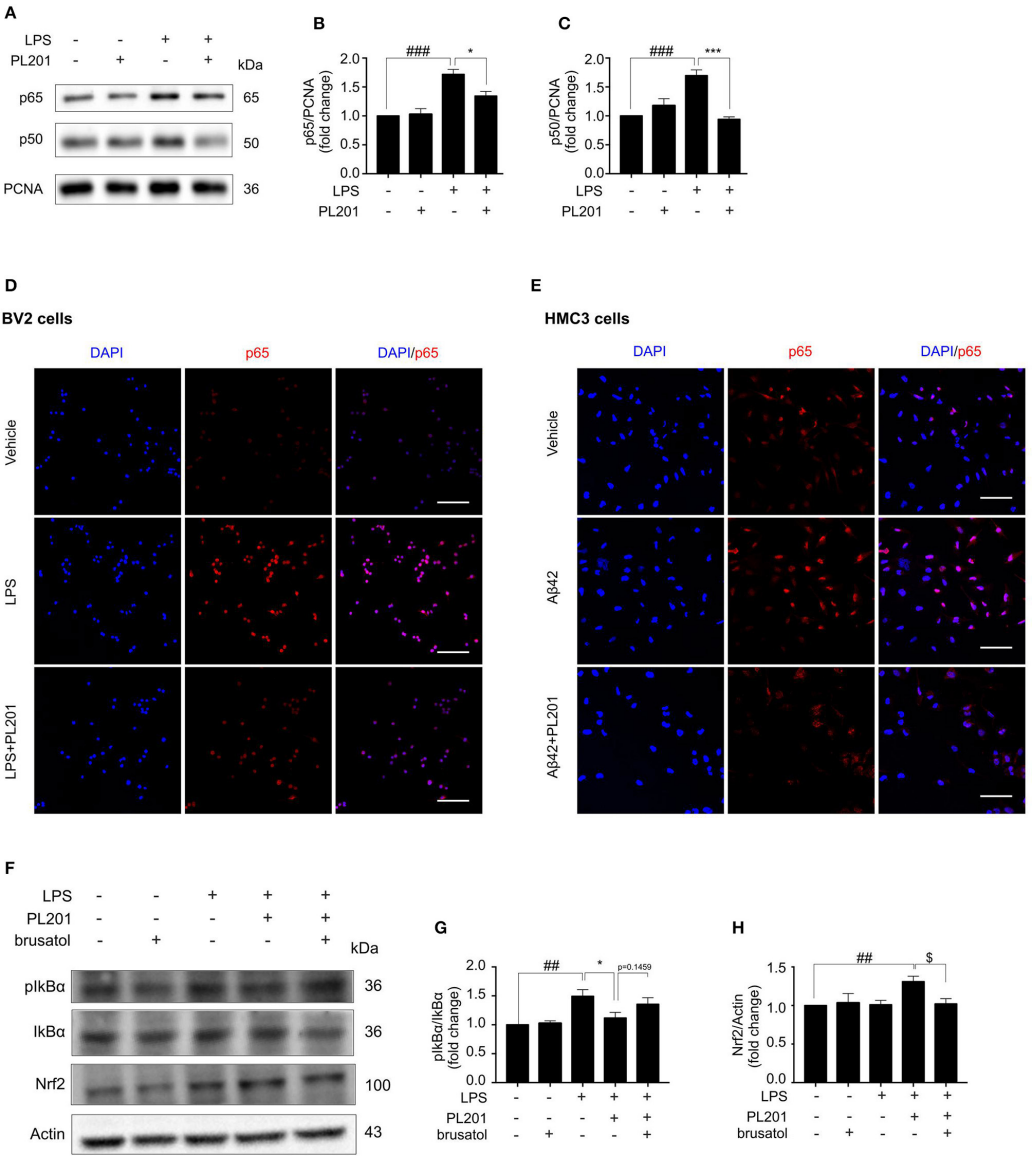
PL201 suppresses the NF-κB pathway. **(A–D**, **F–H)** BV2 cells were pretreated with PL201 for 2 h followed by LPS for further 1 h. **(A–C)** The intranuclear protein expression of p50 and p65 was measured via Western blot. **(D)** The nuclear translocation of p65 in BV2 cells was detected by immunostaining. Scale bar = 100 μm. **(E)** HMC3 cells were pretreated with PL201 for 2 h followed by Aβ42 for further 1 h. The nuclear translocation of p65 in HMC3 cells was detected by immunostaining. Scale bar = 100 μm. **(F–H)** BV2 cells were treated with PL201 in the presence or absence of brusatol (30 nM) and then exposed to LPS for 1 h; the protein levels of phosphorylated IkBα and total IkBα were analyzed by Western blot, the expression of Nrf2 was measured by Western blot. Quantifications were expressed as mean ± SEM (compared with vehicle, ^#^*p* < 0.05, ^##^*p* < 0.01, ^###^*p* < 0.005; compared with LPS-stimulated condition, *p < 0.05, **p < 0.01, ***p < 0.005; compared with PL201 under brusatol treatment, ^$^*p* < 0.05, ^$$^*p* < 0.01, ^$$$^*p* < 0.005).

Emerging evidence suggest that the cross-talk between Nrf2 and NF-κB signaling is important for the regulation of inflammation. To explore the effect of PL201 on NF-κB pathway, BV2 cells were pretreated with PL201 for 2 h followed by LPS stimulation for another 1 h. After that, nuclear protein was extracted as previously reported. The nuclear accumulation of p50 and p65 markedly increased after LPS treatment, which is reduced by PL201 treatment (Figures 5A–C). Using immunofluorescence microscopy, we also observed that PL201 significantly inhibited the nuclear translocation of the NF-κB p65 subunit in LPS or oligomer Aβ42-activated microglia cells (Figures 5D,E). IκB-α is a negative regulator of NF-κB. Previous reports showed that elevating the expression of Nrf2 inhibited NF-κB activity. We hypothesized that PL201 might suppress NF-κB activity by inducing Nrf2. To test this, we pretreated BV2 cells in the absence or presence of brusatol or PL201, followed by LPS stimulation for 1 h, and found that brusatol reversed the inhibitory effect of PL201 on IκB-α phosphorylation, while the basal protein levels of Nrf2 and IκB-α have no changes after brusatol stimulation (Figures 5F–H). These results demonstrated that Nrf2 mediates PL201-dependent downregulation of NF-κB activity.

## Discussion

In AD, microglia accumulate and secrete proinflammatory cytokines, which accelerate neuronal injury (42, 43). Our results demonstrated that PL201 treatment significantly reduced the levels of microglia activation and proinflammatory cytokines in the brains of APP/PS1 mice. Furthermore, we found that PL201 inhibited proinflammatory cytokine production in microglia cells after LPS or Aβ stimulation, which indicated that PL201 may affect microglia directly. In summary, these data demonstrated that PL201 may suppress the inflammatory response mediated by microglia both *in vivo* and *in vitro*.

Nrf2 has been reported to regulate the inflammatory response of microglia, and involved in the pathogenesis of AD (18, 44). Thus, we evaluated whether the effect of PL201 was mediated by Nrf2. The results indicated that PL201 could activate Nrf2 signaling by promoting Nrf2 metastasis into the nuclei of microglia cells. PL201 also elevated the mRNA expression of Nrf2 in a dose-dependent manner. Meanwhile, the expression of Nrf2 target genes concluding HO-1, NQO1, and GCLM could be induced by PL201. Interestingly, the anti-neuroinflammatory effect of PL201 can be blocked by Nrf2 inhibitor. PL201 could inhibit NF-κB signal dependent on Nrf2 activation. Collectively, we proposed that PL201 is a Nrf2 signaling activator. Emerging evidence indicated that Nrf2 may play an important role in tau pathology (27, 45, 46). Maybe PL201 could affect tau phosphorylation. Nrf2 also acts as a regulator of autophagy and takes an important role in regulating AD-related protein (47). Future work will be needed to detect if pharmacological induction of Nrf2 may be a valid strategy to facilitate degradation of toxic proteins such as Aβ or tau in the brain.

A large number of anti-inflammatory drugs, including non-steroidal anti-inflammatory drug (NSAID), peroxisome proliferator-activated receptor gamma (PPAR-γ) activators, and TNFα inhibitors, have been assessed in clinical trials for AD, but the results are not yet conclusive (2, 4). Since the complicated etiology of AD, developing multitarget drugs might be a promising strategy to treat this disease. We previously reported that PL201 treatment improved mitochondrial function of neural stem cells and promoted neurogenesis in AD mice. In this study, we further demonstrated that PL201 stimulates Nrf2 signaling in microglia and significantly suppresses neuroinflammation. Taken together, our results indicated that PL201 may serve as a potential multitarget treatment for AD.

## Conclusions

In summary, we found that PL201 significantly reduced neuroinflammation in microglia via Nrf2/HO-1/NF-κB signaling pathway. Our findings indicated that PL201 might serve as a promising therapeutic agent for AD.

## Data Availability Statement

All datasets generated for this study are included in the article/Supplementary Material.

## Ethics Statement

The animal study was reviewed and approved by the Institutional Animal Care and Use Committee (IACUC) of the Institute of Biochemistry and Cell Biology, Shanghai Institutes for Biological Sciences, Chinese Academy of Sciences.

## Author Contributions

YA designed this research and performed the cellular experiments and wrote the manuscript. YA and HZ conducted the animal experiments. GP and SH revised the manuscript. All authors read and approved the final manuscript.

### Conflict of Interest

The authors declare that the research was conducted in the absence of any commercial or financial relationships that could be construed as a potential conflict of interest.

## Supplementary Material

Figure S1 PL201 has no effects on the expression of anti-neuroinflammatory cytokines. BV2 cells were pretreated with PL201 for 2 h followed by LPS stimulation for further 24 h. mRNA expressions of IL-4 **(A)**, IL-10 **(B)**, and CD206 **(C)** were measured by qRT-PCR. Quantifications were expressed as mean ± SEM.

Figure S2 PL201 attenuates Aβ -induced pro-inflammatory factor release *in vitro*. **(A,B)** PL201 has no cytotoxicity on HMC3 cells. **(C–H)** HMC3 cells were pretreated with PL201 for 2 h followed by Aβ42 stimulation for further 24 h. The mRNA expressions of IL-6, TNFα, MCP-1, IL-1β, iNOS, and COX-2 were measured by qRT-PCR. Quantifications were expressed as mean ± SEM (compared with control: #*p* < 0.05, ##*p* < 0.01, ###*p* < 0.005; compared with Aβ-stimulated condition: ^*^*p* < 0.05, ^**^*p* < 0.01, ^***^*p* < 0.005).

Figure S3 PL201 elevates Nrf2 expression. **(A,B)** BV2 cells and HMC3 cells were treated with PL201 for 1 h, Nrf2 expression was detected by western blot. **(C,D)** BV2 cells and HMC3 cells were treated with PL201 for 1 h, the mRNA expression of Nrf2 was measured by qRT-PCR. **(E,F)** BV2 cells were treated with PL201 in the presence or absence of brusatol (30 nM) for 6 h, the protein level of HO-1 was analyzed by western blot. Quantifications were expressed as mean ± SEM (compared with control: #*p* < 0.05, ##*p* < 0.01, ###*p* < 0.005).

Figure S4 PL201 has no effects on the expression of HO-2 protein. BV2 cells and HMC3 cells were pretreated with PL201 for 6 h. **(A–C)** Representative western blot analysis of HO-2 and mRNA expression of HO-2 in BV2 cells. **(D–F)** Representative western blot analysis of HO-2 and mRNA expression of HO-2 in HMC3 cells. Quantifications were expressed as mean ± SEM.

Figure S5 PL201 activates the expression of GCLM and NQO1. BV2 cells were pretreated with PL201 for 6 h. **(A–C)** Representative western blot analysis of GCLM and mRNA expression of GCLM. **(D–F)** Representative western blot analysis of NQO1 and mRNA expression of NQO1. Quantifications were expressed as mean ± SEM (compared with vehicle: #*p* < 0.05, ##*p* < 0.01, ###*p* < 0.005).

Figure S6 The effect of PL201, brusatol and Snpp on cytokines expression alone. **(A–C)** BV2 cells were treated with PL201, brusatol and Snpp alone stimulation for 24 h. The expressions of IL-6, TNF-α, and IL-1β were detected by qRT-PCR.

## Abbreviations

AD: Alzheimer's disease
Aβ42: amyloid beta42
CNS: central nervous system
DMEM: Dulbecco's minimal essential medium
MEM: minimal essential medium
DMEM/F12: Dulbecco's modified Eagle's medium nutrient mixture F-12
ELISA: enzyme-linked immunosorbent assay
FBS: fetal bovine serum
HFIP: hexafluoroisopropanol
DMSO: dimethyl sulfoxide
LPS: lipopolysaccharides
IL-1β: interleukin-1 beta
IL-6: interleukin-6
TNFα: tumor necrosis factor alpha
MCP-1: monocyte chemoattractant protein
iNOS: inducible nitric oxide synthase
MIP-1α: macrophage inflammatory protein 1alpha
COX-2: cyclooxygenase-2
IL-4: interleukin 4
IL-10: interleukin 10
CD206: cluster of differentiation 206
Iba1: ionized calcium binding adaptor molecule
GFAP: glial fibrillary acidic protein
Nrf2: nuclear factor erythroid 2-related factor
HO-1: heme oxygenase-1
HO-2: heme oxygenase-2
NQO1: NAD(P)H dehydrogenase quinone1
GCLM: glutamate-cysteine ligase modifier
SNPP: Tin protoporphyrin IX
PBS: phosphate-buffered saline
TBS: Tris-buffered saline
DAPI: 4′6-diamidino-2-phenylindole
NF-κB: nuclear factor-kappa B
qRT-PCR: quantitative real-time polymerase chain reaction
RT: room temperature
SDS-PAGE: sodium dodecyl sulfate polyacrylamide gel electrophoresis
IκBα: nuclear factor of kappa light polypeptide gene enhancer in B-cell inhibitor, alpha.
