# Supplementary figures and images for: PL201, a Reported Rhamnoside Against Alzheimer's Disease Pathology, Alleviates Neuroinflammation and Stimulates Nrf2 Signaling
Source: Front Immunol. 2020 Feb 27;11:162. doi: 10.3389/fimmu.2020.00162 (PMC7056876; doi:10.3389/fimmu.2020.00162)

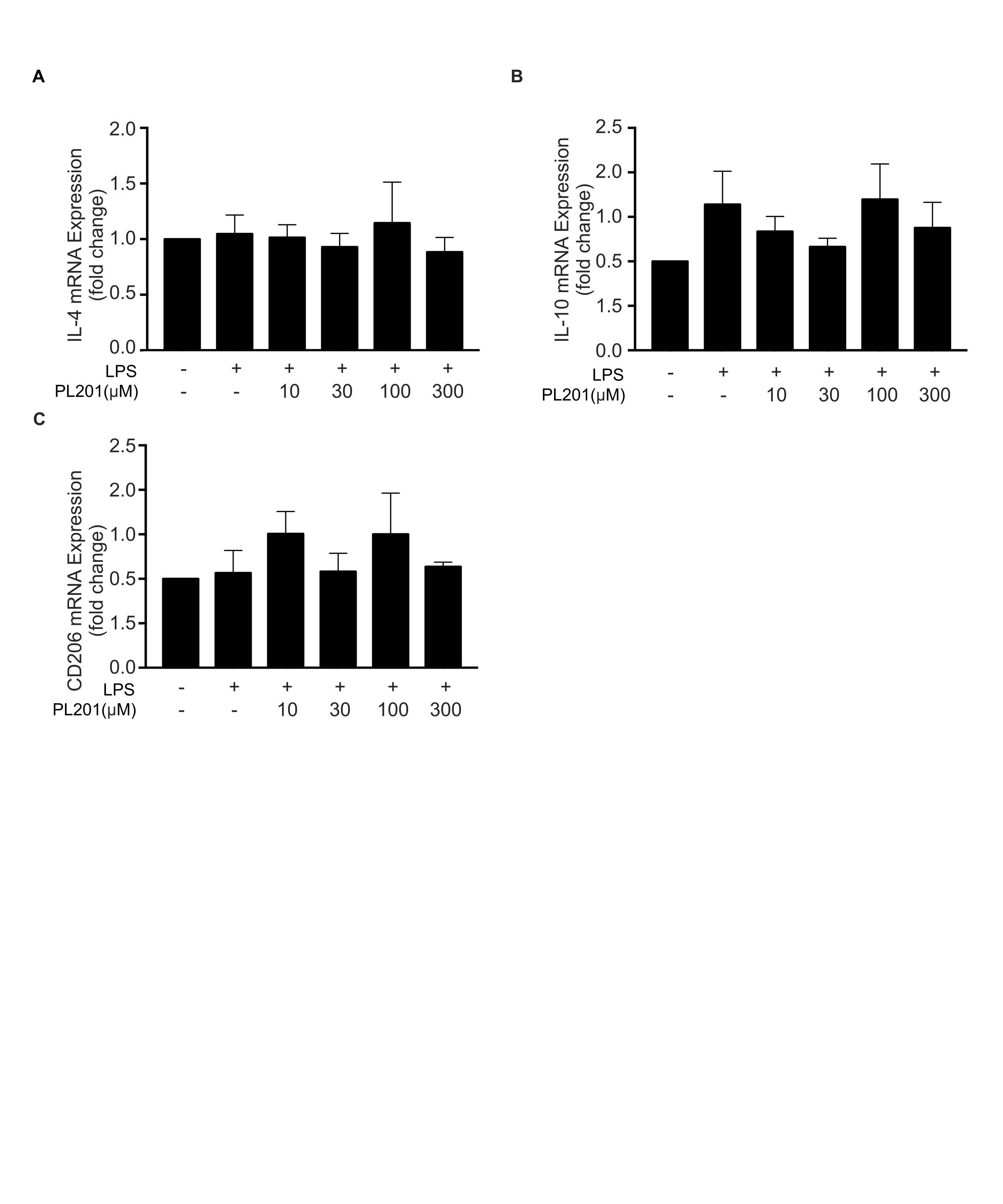

Supplement: Figure S1 — PL201 has no effects on the expression of anti-neuroinflammatory cytokines. BV2 cells were pretreated with PL201 for 2 h followed by LPS stimulation for further 24 h. mRNA expressions of IL-4 (A), IL-10 (B), and CD206 (C) were measured by qRT-PCR. Quantifications were expressed as mean ± SEM. [file Image_1.jpg]

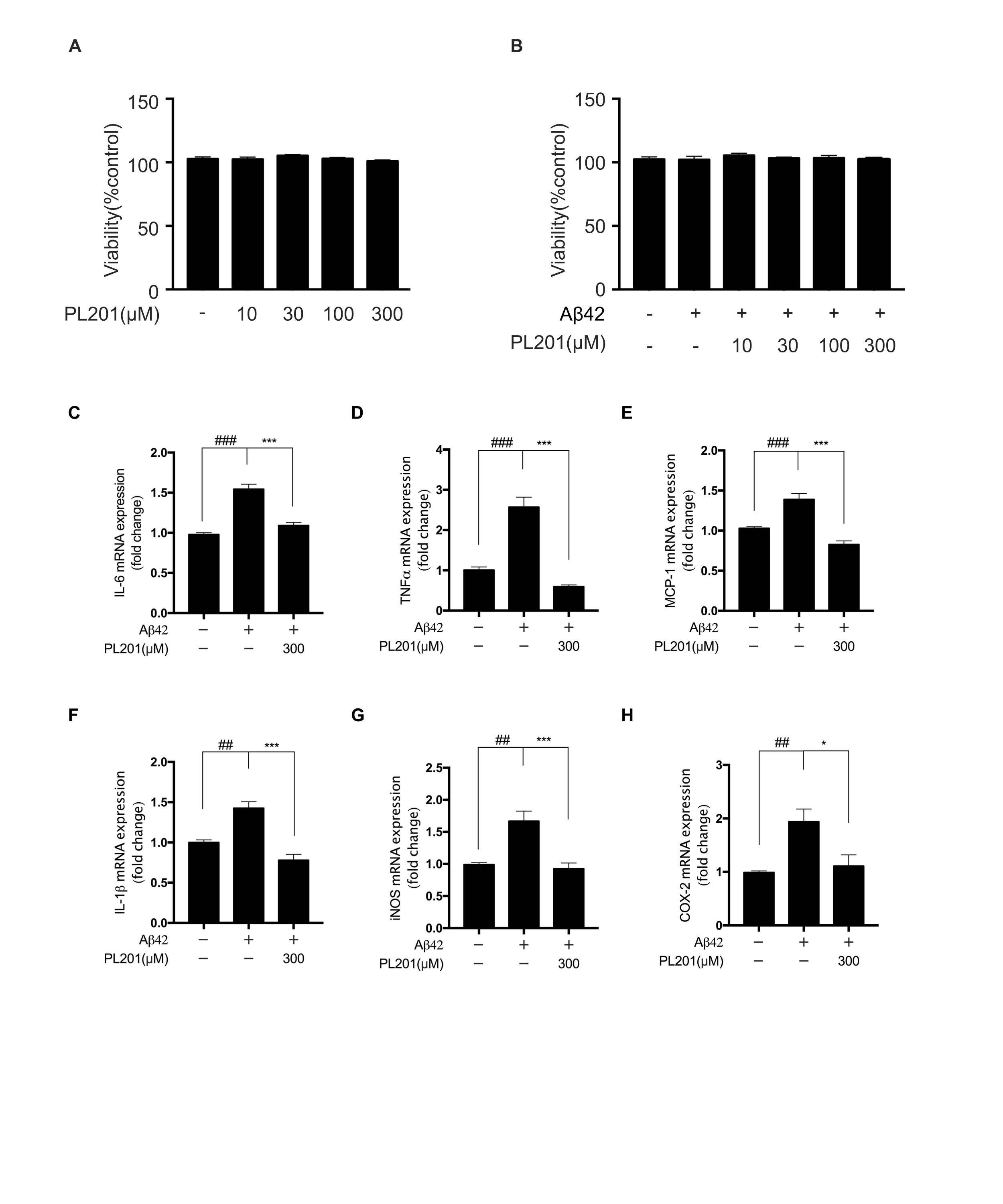

Supplement: Figure S2 — PL201 attenuates Aβ -induced pro-inflammatory factor release in vitro. (A,B) PL201 has no cytotoxicity on HMC3 cells. (C–H) HMC3 cells were pretreated with PL201 for 2 h followed by Aβ42 stimulation for further 24 h. The mRNA expressions of IL-6, TNFα, MCP-1, IL-1β, iNOS, and COX-2 were measured by qRT-PCR. Quantifications were expressed as mean ± SEM (compared with control: #p < 0.05, ##p < 0.01, ###p < 0.005; compared with Aβ-stimulated condition: *p < 0.05, **p < 0.01, ***p < 0.005). [file Image_2.jpg]

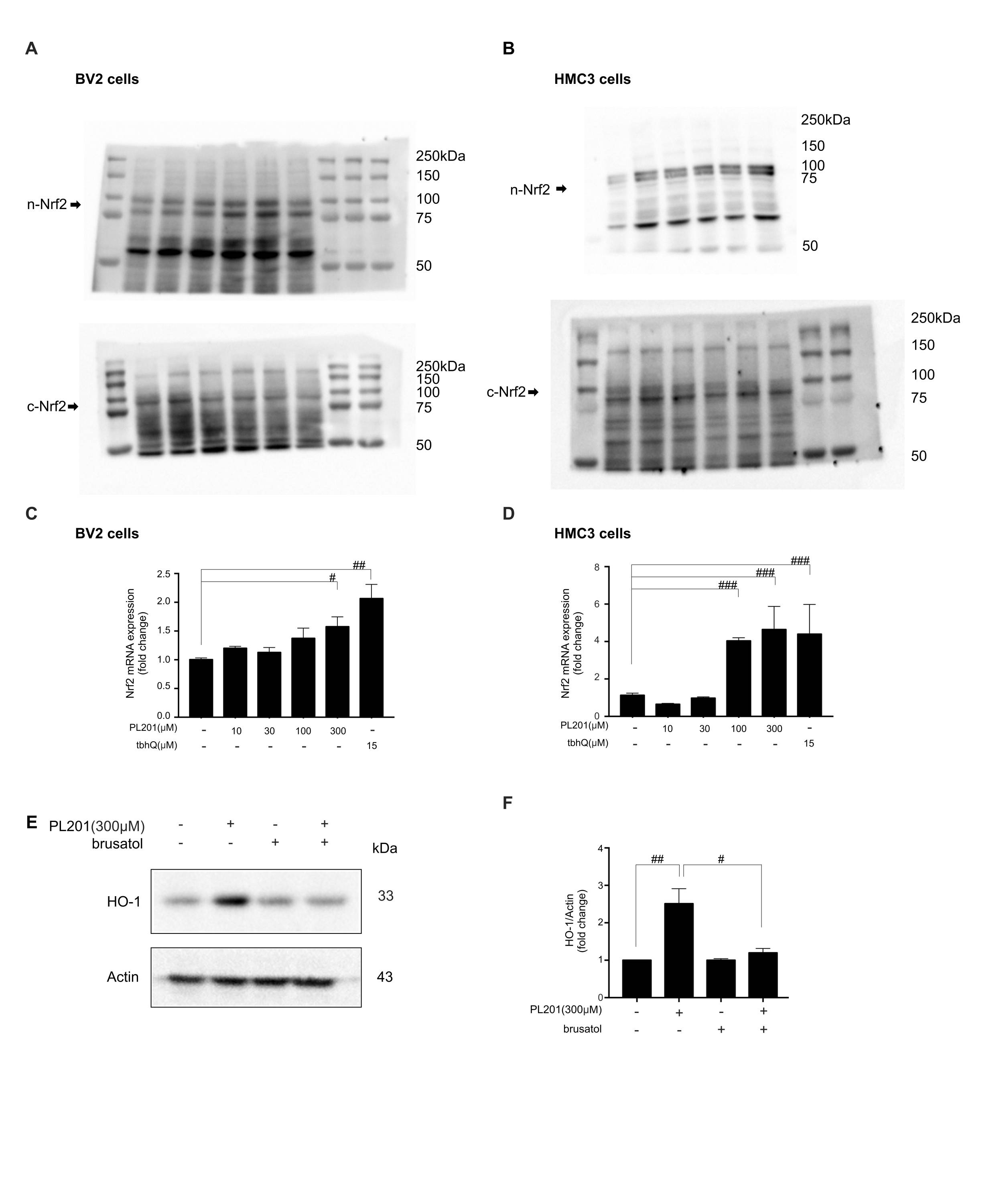

Supplement: Figure S3 — PL201 elevates Nrf2 expression. (A,B) BV2 cells and HMC3 cells were treated with PL201 for 1 h, Nrf2 expression was detected by western blot. (C,D) BV2 cells and HMC3 cells were treated with PL201 for 1 h, the mRNA expression of Nrf2 was measured by qRT-PCR. (E,F) BV2 cells were treated with PL201 in the presence or absence of brusatol (30 nM) for 6 h, the protein level of HO-1 was analyzed by western blot. Quantifications were expressed as mean ± SEM (compared with control: #p < 0.05, ##p < 0.01, ###p < 0.005). [file Image_3.jpg]

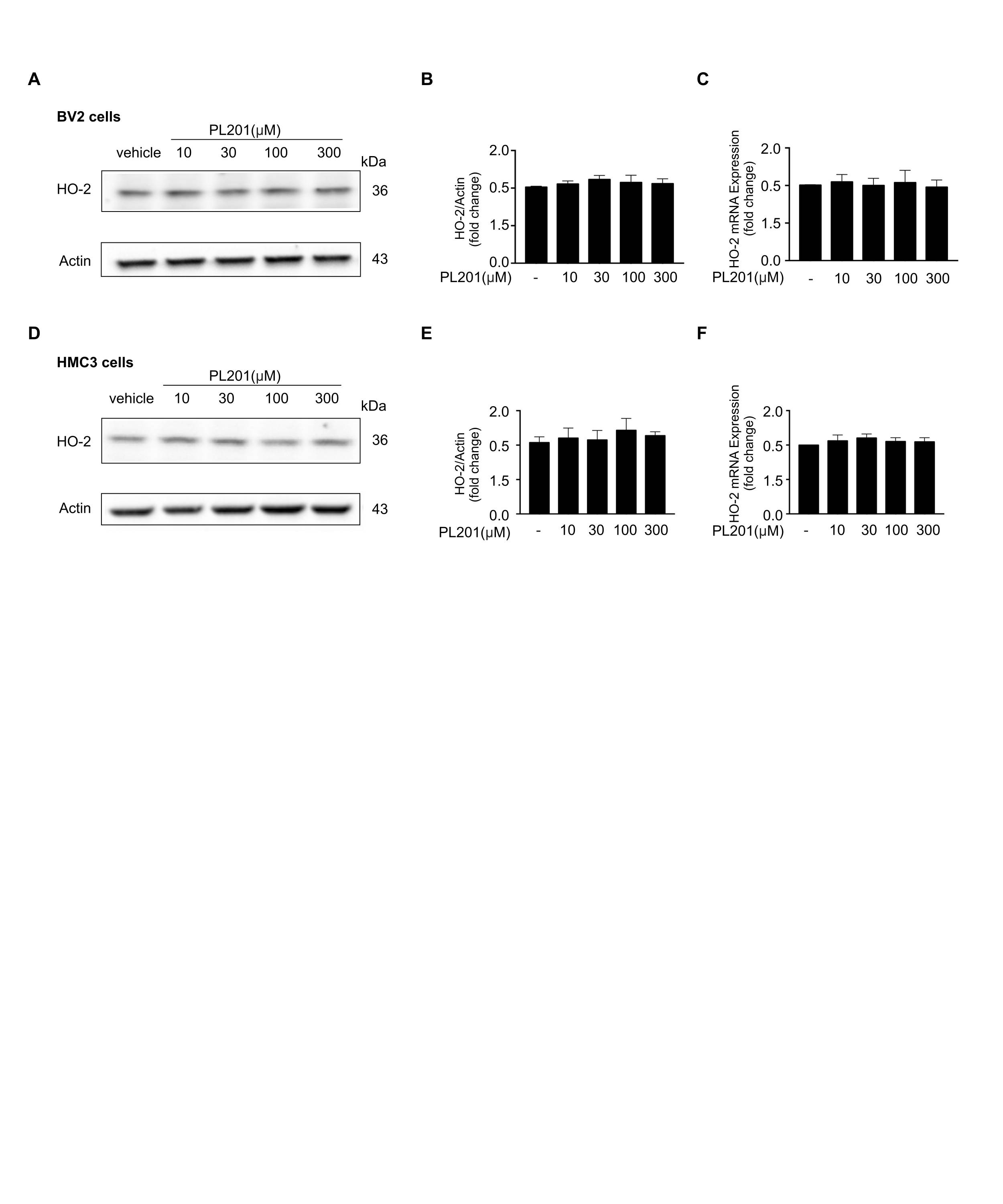

Supplement: Figure S4 — PL201 has no effects on the expression of HO-2 protein. BV2 cells and HMC3 cells were pretreated with PL201 for 6 h. (A–C) Representative western blot analysis of HO-2 and mRNA expression of HO-2 in BV2 cells. (D–F) Representative western blot analysis of HO-2 and mRNA expression of HO-2 in HMC3 cells. Quantifications were expressed as mean ± SEM. [file Image_4.jpg]

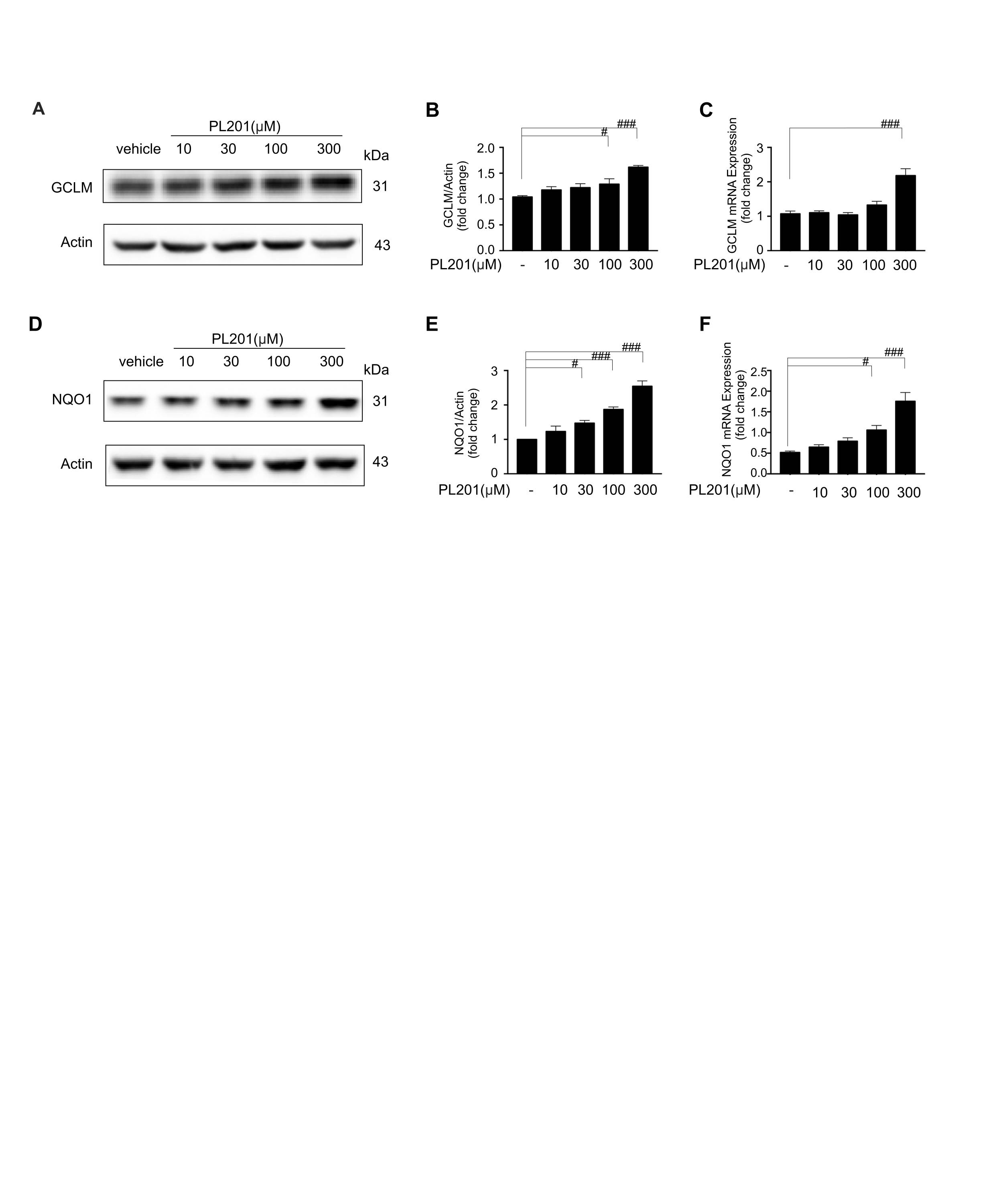

Supplement: Figure S5 — PL201 activates the expression of GCLM and NQO1. BV2 cells were pretreated with PL201 for 6 h. (A–C) Representative western blot analysis of GCLM and mRNA expression of GCLM. (D–F) Representative western blot analysis of NQO1 and mRNA expression of NQO1. Quantifications were expressed as mean ± SEM (compared with vehicle: #p < 0.05, ##p < 0.01, ###p < 0.005). [file Image_5.jpg]

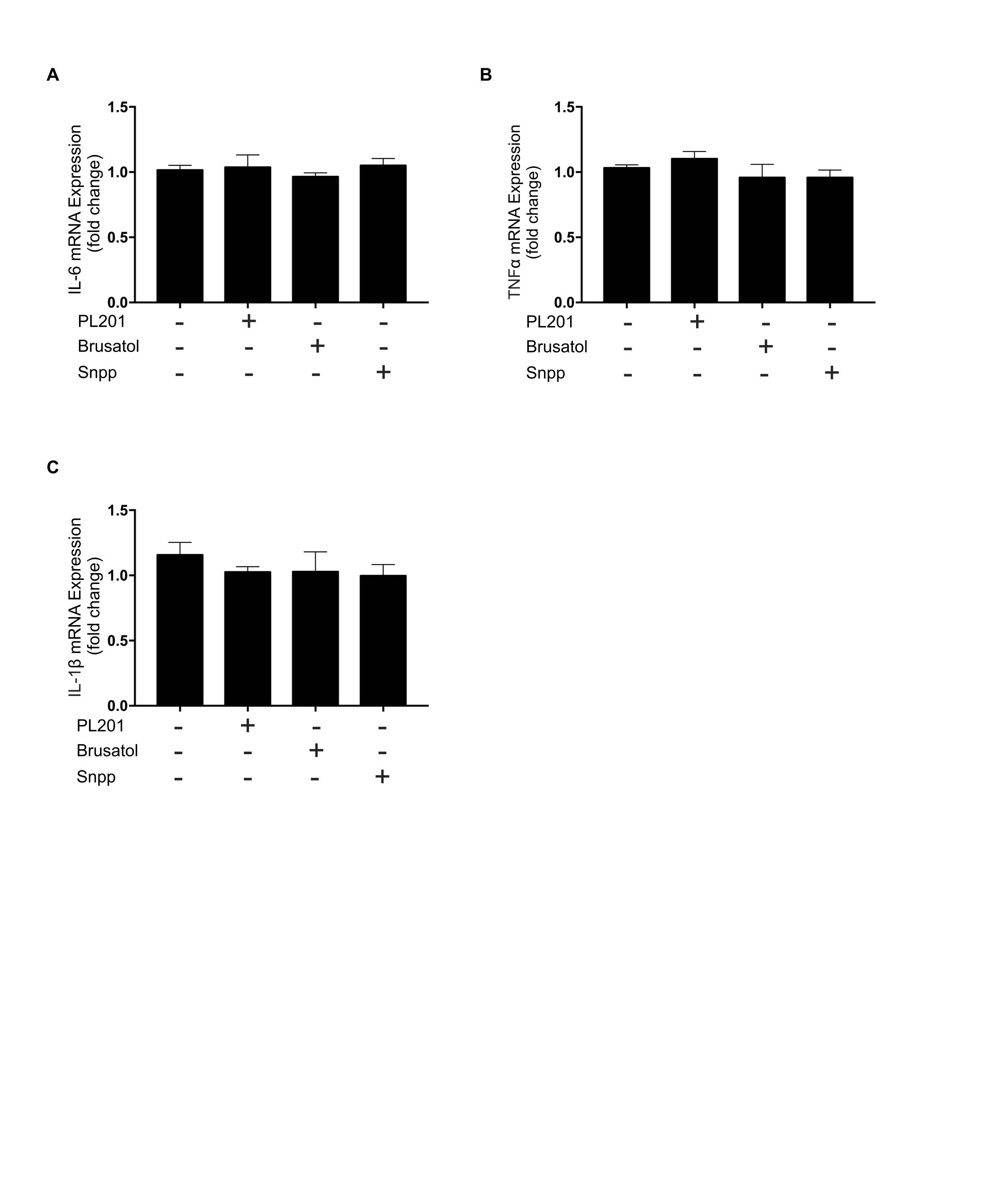

Supplement: Figure S6 — The effect of PL201, brusatol and Snpp on cytokines expression alone. (A–C) BV2 cells were treated with PL201, brusatol and Snpp alone stimulation for 24 h. The expressions of IL-6, TNF-α, and IL-1β were detected by qRT-PCR. [file Image_6.jpg]
